# Studying tourism development and its impact on carbon emissions

**DOI:** 10.1038/s41598-024-58262-w

**Published:** 2024-03-29

**Authors:** Xiaochun Zhao, Taiwei Li, Xin Duan

**Affiliations:** School of Management, Anhui University, Hefei, China

**Keywords:** Tourism industry, Carbon emission efficiency, Influence mechanism, Structural equation model, Environmental impact, Sustainability

## Abstract

Analyzing the influence of tourism on carbon emission has significant implications for promoting the sustainable development of tourism. Based on the panel data of 31 tourist cities in China from 2005 to 2022, this study utilizes a structural equation model to explore the carbon reduction effect of tourism development and its influencing mechanism. The results show that: (1) The overall carbon emission efficiency of tourism cities first decreased and then increased, rised to a peak of 0.923 in 2022. (2) Tourism development has a significant positive impact on carbon emission efficiency, and there are three influence paths: tourism → environmental regulation → carbon emission efficiency, tourism → environmental regulation → industrial structure → carbon emission efficiency, and tourism → industrial structure → carbon emission efficiency. (3) The influence of tourism development on carbon emission efficiency mainly depends on the direct effect, and the development of tourism also indirectly affect the industrial structure. Environmental regulation also mainly depends on the direct effect on carbon emission efficiency. (4) Foreign direct investment lead to the reduction of carbon emission efficiency in both direct and indirect aspects.

## Introduction

Global climate change has become one of the major challenges of humanity, bringing a series of harms, including an increase in extreme weather events such as heatwaves, droughts, floods, and hurricanes. According to the report of United Nations Intergovernmental Panel on Climate Change (IPCC), by 2080, the average global temperature will increase by more than 1 °C^1^. Global warming is not merely a natural phenomenon, but also a result of human activity. In various sectors of the economy, the tourism industry has experienced rapid growth. According to data from the World Tourism Organization, the tourism industry accounts for 10.4% of the global Gross Domestic Product (GDP) and provides 313 million job opportunities^2^. However, the rapid development of the tourism industry has also resulted in intensified impacts on the environment. Tourism industry has become one of the main sources of global carbon dioxide emissions, accounting for 5% of the total global carbon emissions. China is one of the largest tourism markets in the world, and the tourism industry plays a vital role in China's economy. In China's 14th Five-Year Plan, the concept of green and low-carbon development is emphasized, highlighting the need for environmentally friendly tourism and carbon emission reduction. Balancing tourism industry development with carbon emission reduction is a major challenge for the tourism industry. Existing studies on tourism and carbon emissions mainly focus on the carbon emission efficiency of tourism development itself and the impact of tourism on carbon emissions. However, these studies fail to analyze the mechanism behind tourism's impact on carbon emission efficiency. While some studies have analyzed the impact mechanism of tourism development and carbon emissions^3^, they primarily focused on the impact of tourism on carbon emission intensity rather than carbon efficiency. Carbon intensity is typically measured as a ratio of carbon emissions to GDP. In contrast, carbon efficiency provides a more comprehensive assessment of a city's environmental performance and sustainability. Comprehensive analyzing the mechanism of the influence of tourism development on carbon emission efficiency is essential for formulating environmental protection policies to promote the green development of tourism. Therefore, this paper aims to study the influence mechanism of tourism on carbon emission efficiency s of tourism development. Using 31 tourist cities in China as research samples, the paper adopts the entropy weight method and the Slacks-Based Measure (SBM), introduces the Structural Equation Model (SEM), and uses panel data of 31 tourist cities to analyze the influence of tourism industry on carbon emission. The findings of this study are hoped to provide inspiration for the transformation of tourism cities.

The remainder of this study is divided into four sections. The first section is the literature review, which examines the carbon emission efficiency of the tourism industry itself and the impact of tourism development on carbon emission efficiency from a tourism research perspective. The second section is the research design, where the paper utilizes the entropy weight method and the super-efficiency SBM model to measure the development level of the tourism industry and carbon emission efficiency, respectively. This section also constructs a structural equation model to explore the mechanism of the impact of tourism development on carbon emission efficiency. The third section presents the research results and the last section concludes this study and provides suggestions based on research findings.

## Literature review

Tourism plays a vital role in economic growth by creating jobs^4^. Scholars have conducted extensive research and achieved significant academic results. The current research on the tourism industry and carbon emission efficiency primarily revolves around two aspects.

Firstly, scholars focus on the carbon emission efficiency of the tourism industry. For example, Gössling et al.^5^ analyzed the economic benefits and environmental effect of tourism, evaluating the ecological efficiency of the tourism industry by using carbon dioxide emissions and economic benefits. Osorio et al.^6^ compared the carbon emission efficiency of the Spanish tourism industry before and after the pandemic of COVID-19, and found that the carbon emission efficiency in 2020 improved compared before COVID-19 pandemic. Ghaderi et al.^7^ conducted research on the carbon emission efficiency of tourism industry in the Middle East and North Africa, this study indicated that tourist arrivals can reduce carbon emissions, while energy consumption and trade openness are contributors to carbon emissions.

Secondly, scholars focus on the impact of tourism on carbon emission. However, the consensus among scholars has not yet been reached on whether the tourism industry promotes carbon emission. Some scholars have analyzed the impact of tourism activities on carbon emissions in Mediterranean countries and concluded that tourism revenue does not have direct impact on carbon emissions^8^. Voumik et al.^9^ studied tourism industry in 40 Asian countries and found that while tourism helps slow down the deterioration of environment, factors such as population growth, energy use, and economic development still contribute to increasing carbon emission, which is consistent with the conclusions of Guo et al.^10^. Erdoğan et al.^11^ focused on the impact of international tourism on carbon emissions and found that international tourism leads to the increase of carbon emissions, but eco-friendly innovation in the transportation sector can mitigate the negative impact on the environment. Ahmad et al.^12^ revealed an inverted U-shaped curve in the impact of international tourism development on carbon emissions in China, with the negative impact of technological innovation being strongest in highly developed provinces and weakest in moderately developed provinces. Ghosh et al.^13^ found tourism industry can alleviate environmental degradation, policy direction that promote tourism, renewable energy, economic growth and urbanization have a significant effect on the environment, which is consistent with the conclusion of Zikirya et al.^14^. Rahman et al.^15^ shifted their focus to Malaysia and found a positive correlation between the number of tourists and carbon emissions.

In summary, existing studies primarily focus on the carbon emission efficiency of tourism and the impact of tourism on carbon emission. However, there is a lack of focus on how tourism affects carbon emission efficiency. This study aims to address this gap by taking 31 tourist cities in China as research samples. This study constructs an indicator system to assess tourism industry and carbon emission efficiency. Furthermore, this study introduces a structural equation model to analyze the mechanisms about how tourism industry affects carbon emission efficiency, to provide inspiration for promoting the green development of tourism.

## Research Design

### Analyzing the influence mechanism of tourism on carbon emission

The tourism industry is considered a smokeless and green industry, due to its significant advantages in resource utilization and environmental protection. The development of the tourism industry can not only promote the growth of employment rate in the destination, but also increase the income of tourist destinations. Compared with the secondary industry, the tourism industry is more environmentally friendly in terms of resource consumption and pollution emission. Especially in tourism cities, the proportion of tourism economy in GDP is larger, tourism has a bigger impact on the green development of the tourism city. Consequently, the influence mechanism of tourism on carbon emissions is analyzed as follows:

Firstly, a good natural ecological environment is a fundamental requirement for the development of the tourism industry. Tourism cities typically implement strict governance measures on the local environment and ecology. The development of tourism can incentive the local government to introduce more stringent environmental policies, thereby improving the ecological environment^16^. Additionally, stricter environmental regulations often impact carbon emission efficiency^17,18^. Simultaneously, intensified environmental regulations can limit the development space of heavily polluting industries and influence the industrial structure of the destination, ultimately affecting carbon emission efficiency^19^.

Secondly, the development of tourism also affects the industrial structure of cities^20^. The growth of tourism promotes the rise of related industries. Numerous supporting industries, such as hotels, catering, transportation, tour guides, and others, are needed to meet the demands of tourists and create numerous employment opportunities. Consequently, tourism development can attract individuals to switch from other industries to the tourism sector, which in turn impacts the industrial structure and has a significant effect on carbon emissions^21^.

Finally, foreign direct investment is an important factor that affects carbon emission efficiency^22^. This study draws on the conclusion of Bakhsh et al.^23^, which suggests that including foreign direct investment in analysis can improves the overall fit of the structural equation model. On one hand, foreign direct investment can bring advanced production technology, thereby directly improving carbon emission efficiency^24^. On the other hand, foreign investment also leads to pollution transfer, negatively impacting the environment and reducing carbon emission efficiency^25^. At the same time, foreign direct investment can indirectly affect carbon emission efficiency by influencing the local industrial structure. Moreover, the advanced technologies brought about by foreign direct investment also have an impact on technological innovation, thereby indirectly affecting carbon emissions.

**Figure 1:**
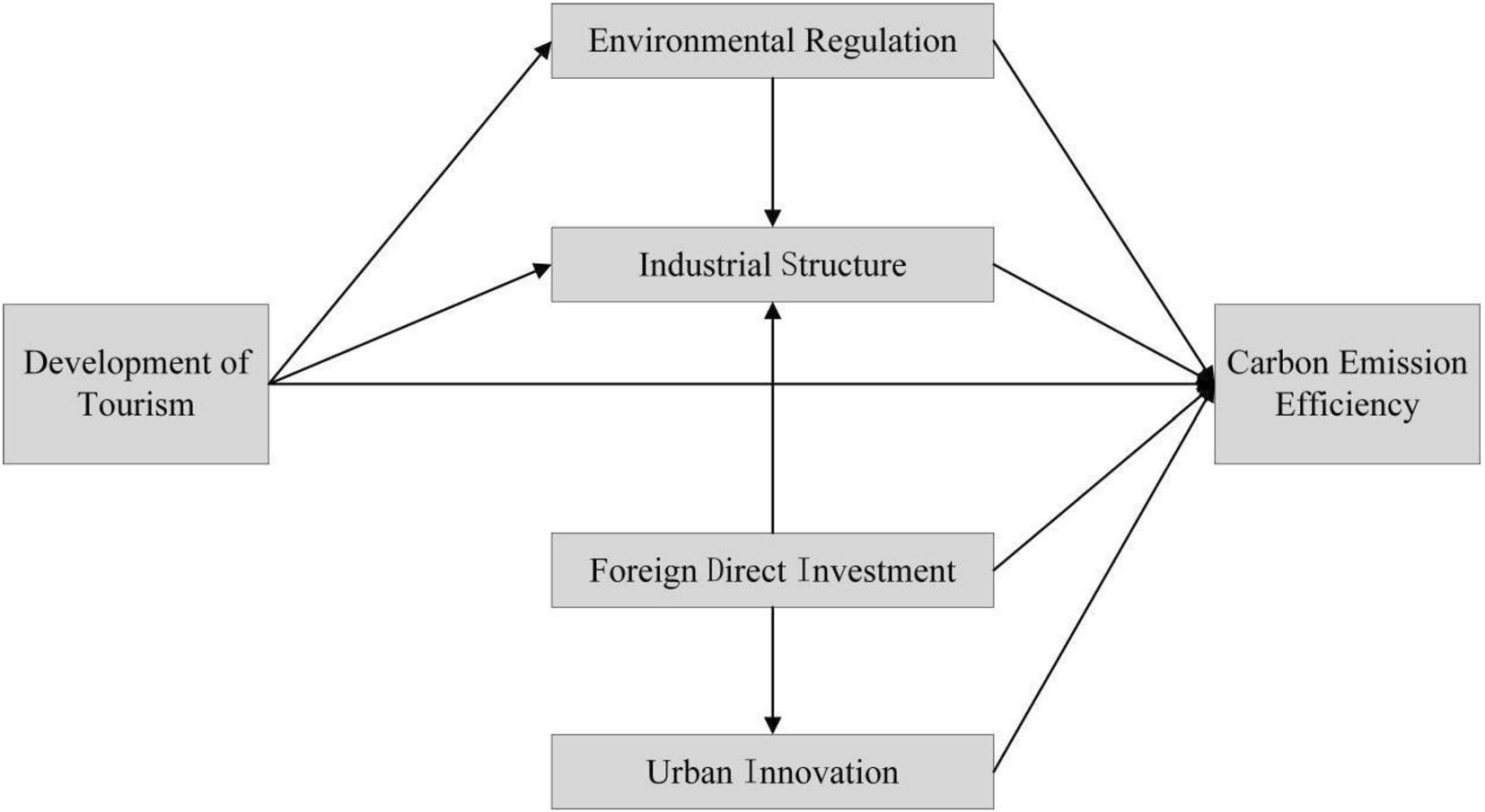
The influence mechanism of tourism on carbon emission.

Based on above analysis, this study builds a structural equation model about the influence of tourism on carbon emission (see Fig. 1).

### Research method

#### Structural equation model

The Structural Equation Model (SEM), first proposed by Jöreskog^26^, is used to study complex relationships among different variables, including multiple causal relationships. When examining the impact of tourism on carbon emissions, it is important to consider that this impact is not a single direct effect. Instead, there are complex internal mechanisms, including indirect effects and interactions among variables^27^. Therefore, this study chooses to employ SEM to analyze the internal mechanism of how tourism affects carbon emission efficiency.

#### Entropy weight method

In this study, the entropy weight method is utilized to calculate the Tourism Development (TD) level. The entropy weight method is a quantitative approach based on the concept of entropy in information theory. It helps determine the weight of indicators by calculating the entropy and difference coefficient of each index. This calculation process reflects the importance of each index in the overall assessment. By multiplying and summing the standardized index with the entropy weight, the assessment results can be obtained^28^. The specific calculation process is as follows:

Firstly, the raw data needs to be standardized, see formula (1) and formula (2) for details.

Positive indicator:

1 $$r_{ij}^{ + } = \frac{{x_{ij} - \min \left( {x_{ij} } \right)}}{{max\left( {x_{ij} } \right) - min\left( {x_{ij} } \right)}}$$

Negative indicator:

2 $$r_{ij}^{ - } = \frac{{\max \left( {x_{ij} } \right) - x_{ij} }}{{max\left( {x_{ij} } \right) - min\left( {x_{ij} } \right)}}$$

Among them,${ }x_{ij} { }$ represents the data of the indicator, $i{ }$ represents city. $j{ }$ represents index,$r_{ij}^{ + }$ and $r_{ij}^{ - }$ represents standardized data.

Secondly, calculate the weight of $j$ index by using formula (3).

3 $$P_{ij} = \frac{{r_{ij} }}{{\mathop \sum \nolimits_{i = 1}^{n} r_{ij} }}$$

Thirdly, calculate the entropy of $j$ by using formula (4).

4 $$E_{j} = - k\mathop \sum \limits_{i = 1}^{n} P_{ij} ln\left( {P_{ij} } \right) k = - \frac{1}{ln\left( n \right)} > 0$$

Fourthly, calculate information entropy redundancy by using formula (5).

5 $$d_{j} = 1 - E_{j} j = 1,2 \ldots ,n$$

Fifthly, calculate index weight by using formula (6).

6 $$W_{j} = \frac{{d_{j} }}{{\mathop \sum \nolimits_{j = 1}^{n} \left( {d_{j} } \right)}}$$

Finally, calculate the assess results by using formula (7).

7 $$S_{i} = \mathop \sum \limits_{j = 1}^{n} W_{j} r_{ij}$$

#### Non-expected output super efficiency SBM model

Tone^29^ proposed a super-efficient model based on the traditional SBM model, which combines the advantages of both the traditional SBM model and the super efficiency model. This model not only considers the influence of unexpected output, but also solves the problem that the traditional SBM model cannot evaluate the Decision-Making Unit (DMU) with the efficiency value of 1 on the front plane. By recalculating the DMUs with an efficiency value of 1, the model enables the comparison of effective DMUs. The specific formulas are as follows:

8 $$\rho^{*} = min\frac{{\frac{1}{m}\mathop \sum \nolimits_{i = 1}^{m} \left( {\frac{{\overline{x}}}{{x_{ik} }}} \right)}}{{\frac{1}{{r_{1} + r_{2} }} \times \left( {\mathop \sum \nolimits_{s = 1}^{{r_{1} }} \frac{{\overline{{y^{d} }} }}{{y_{sk}^{d} }} + \mathop \sum \nolimits_{q = 1}^{{r_{2} }} \frac{{\overline{{y^{u} }} }}{{y_{qk}^{u} }}} \right)}}$$

9 $$\begin{array}{*{20}c} {\overline{x}\geq\mathop \sum \limits_{j = 1, \ne k}^{n} x_{ij} \sigma_{j} \left( {i = 1,2, \ldots ,m} \right)} \\ {\overline{{y^{d} }}\leq \mathop \sum \limits_{j = 1, \ne k}^{n} y_{sj}^{d} \sigma_{j} \left( {s = 1,2, \ldots ,r_{1} } \right)} \\ {\overline{{y^{u} }}\geq \mathop \sum \limits_{j = 1, \ne k}^{n} y_{q}^{u} \sigma_{j} \left( {q = 1,2, \ldots ,r_{2} } \right)} \\ {\sigma_{j} 0\left( {j = 1,2, \ldots ,n} \right)} \\ {\overline{x}x_{ik} \left( {j = 1,2, \ldots ,m} \right)} \\ {\overline{{y^{d} }} y_{sk}^{d} \left( {s = 1,2, \ldots ,r_{1} } \right)} \\ {\overline{{y^{u} }} y_{q}^{u} \left( {u = 1,2, \ldots ,r_{2} } \right)} \\ \end{array}$$

### Designing index system for tourism and carbon emission efficiency, variable explaining and data source

#### Designing index system and variable interpretation

This study utilizing the entropy weight method to calculate the Tourism Development level(TD). To evaluate the development level of tourism, this paper designs the index system of tourism development (see Table 1). Firstly, the number of tourists is an important indicator that represents the development of tourism, as it reflects the scale of tourism and market demand^30^. Secondly, tourism income is a crucial index for measuring the economic benefit of tourism, as it represents the economic benefit and profit level of tourism. Tourism income directly impacts the sustainable development of tourism and related industries. Finally, the proportion of tourism revenue to GDP is an essential indicator for measuring the contribution and impact of tourism on the overall economy. On the basis of previous studies, this study constructs the evaluation index system of tourism development level.

**Table 1:** Tourism development level measurement index system.

Variable	Evaluation index	P/N
Development of tourism	Number of domestic tourists (10,000)	+
	Domestic tourism revenue (100 million yuan)	+
	Number of foreign tourists (10,000)	+
	Foreign tourism revenue (100 million yuan)	+
	Tourism revenue as a percentage of GDP (%)	+

The essence of Carbon Emission Efficiency (CEE) is the result of the joint action of capital, labor, energy, and other inputs and outputs in economic activities. Therefore, adopting a multi-input and multi-output perspective, this study uses MATLAB software to measure the carbon emission efficiency of 31 tourist cities. Acknowledging that efficiency values are influenced by both inputs and outputs, this study selects five indicators: labor input, capital input, energy input, expected output, and undesirable output to measure carbon emission efficiency (see Table 2). Firstly, the total number of employees in enterprises and public institutions reflects the economic scale of state-owned enterprises and public institutions, while the total number of urban private self-employed employees highlights the scale of the development of the private and individual economy^31^. Therefore, the sum of the total number of employees in enterprises and public institutions and the total number of private and individual employees in cities and towns is chosen as the representative of labor input, which fully reflects the employment scale and labor supply of a country or region. Secondly, electricity is widely used as an energy source in cities, and its consumption largely reflects a city's energy consumption^32^. The total electricity consumption of the city is selected to represent the energy input. Thirdly, investments in fixed assets reflects the investment of a country or region in capital goods such as production equipment and buildings over a certain period, and it is an important measure of capital formation^33^. The capital stock of the city is calculated based on the investment in fixed assets to represent the capital input. Fourthly, GDP is the sum of all the market value created by all the residents of a country or region in a certain period, and it is the most important macroeconomic indicator for measuring the overall economic performance of a country or region^34^. Fifthly, undesirable outputs usually denote by-products or negative effects that occur during the production process, which are not the desired outcomes of manufacturing activities. Carbon dioxide emissions are selected as the undesirable outputs. Finally, this paper takes 2005 as the base period to calculate the capital stock and GDP, to enhance the comparability of data between different years.

**Table 2:** Carbon emission efficiency measurement index system of tourist city.

Indicators	Primary index	Secondary index (unit)
Input indicators	Capital Investment	Capital stock (100 million yuan)
	Labor input	Number of employees (10,000)
	Energy input	Annual electricity consumption (10,000 kwh)
Output indicators	Expected output	Real GDP (100 million yuan)
	Undesirable-outputs	CO_2_ emissions (billion tons)

#### Variables involved in structural equation model

Based on the existing research and data availability, proxy variables for the structural equation model are set up (see Table 3). (1) Tourism, calculated by entropy weight method, reflects the development level of urban tourism; (2) Carbon emission efficiency, calculated by the non-expected output super efficiency SBM, reflects the carbon emission and resource utilization efficiency of the city; (3) Environmental regulation. Currently, there are three quantitative methods for environmental regulation, which are single index method^35^, scoring method^36^ and comprehensive index method^37^. This paper uses the proportion of investment in environmental pollution control in GDP(Gross Domestic Product) as a proxy variable for environmental regulation. (4) Industrial structure, the proportion of the output value of the tertiary industry and the output value of the secondary industry are used as the proxy variable,(5) Foreign direct investment, some scholars believe that foreign direct investment has a negative impact on the environment, supporting the pollution paradise hypothesis, while other scholars believe that foreign direct investment has an improving effect on the environment, supporting the pollution halo hypothesis. Because of fact that the stock of foreign investment can more accurately reflect the impact of foreign investment on environmental pollution, this paper adopts the proportion of foreign direct investment in regional GDP as a proxy variable by referring to the practice of Afi et al.^38^. (6) Urban innovation, referring to the research of Cheng et al.^39^, China's urban innovation index is adopted as a proxy variable. The index is mainly based on two parts of data, namely patent data of the State Intellectual Property Office and enterprise registered capital data of the State Administration for Industry and Commerce, including innovation output and patent value.

**Table 3:** Structural equation model variable description.

Variable	Proxy variable (symbol)
Tourism Development (TD)	Logarithm of the level of urban tourism development (ln *tour*)
Carbon Emission Efficiency(CEE)	Logarithm of a city's carbon efficiency (ln *cee*)
Environmental Regulation(ER)	The proportion of investment in environmental pollution control in GDP (ln *envi*)
Industrial Structure(IND)	The ratio of the output value of the tertiary industry to the output value of the secondary industry (ln *ind*)
Foreign Direct Investment(FDI)	Logarithm of foreign direct investment as a share of GDP (ln *fdi*)
Urban Innovation(INNOV)	The logarithm of the city's innovation composite index (ln *innov*)

#### Research sample and data source

This study selects Chinese tourism cities as research samples to explore the influence of tourism on carbon emission efficiency. This study refers to the research of Zhang et al.^40^ and Huang et al.^41^, a total of 31 tourism cities were selected as research samples. These cities include Beijing, Tianjin, Shenyang, Dalian, Shanghai, Nanjing, Wuxi, Suzhou, Hangzhou, Ningbo, Xiamen, Jinan, Qingdao, Guangzhou, Shenzhen, Zhuhai, Zhongshan, Guilin, Haikou, Wenzhou, Changchun, Harbin, Huangshan, Wuhan, Changsha, Luoyang, Zhangjiajie, Chongqing, Chengdu, Kunming and Xi 'an.

The study period was from 2005 to 2022. The data in this study were obtained from China Urban Statistical Yearbook (2006–2022), China Energy Statistical Yearbook (2006–2022), statistical yearbooks and statistical bulletins of provinces and cities.

## Research results

### Evaluation results of carbon emission efficiency and tourism development in tourist cities

This study measured the carbon emission efficiency of 31 tourist cities from 2005 to 2022 and revealed its evolution characteristics. The calculation results are shown in Table 4.

**Table 4:** Carbon emission efficiency and tourism development of tourist cities.

Year	TD	CEE	Year	TD	CEE
2005	0.125	0.756	2014	0.303	0.835
2006	0.147	0.715	2015	0.329	0.847
2007	0.164	0.741	2016	0.370	0.822
2008	0.169	0.726	2017	0.417	0.833
2009	0.183	0.723	2018	0.448	0.862
2010	0.224	0.707	2019	0.499	0.864
2011	0.249	0.716	2020	0.228	0.920
2012	0.277	0.810	2021	0.197	0.920
2013	0.283	0.834	2022	0.147	0.923

According to Table 4, a clear upward trend is evident in the tourism development level of 31 tourist cities from 2005 to 2019, with the level increasing from 0.125 in 2005 to 0.499 in 2019, thereby reaching its peak. From 2020 to 2022, due to the impact of COVID-19, the number of tourists decreased, and the development level of tourism dropped significantly. From 2005 to 2022, the carbon emission efficiency of 31 tourism cities generally showed a fluctuating upward trend. The overall efficiency decreased year by year from 2005 to 2011, reaching its lowest at 0.707. But then it began to fluctuate and rise and reached a peak of 0.923 in 2022.

### Analyzing the influence of tourism development on carbon emission efficiency and its influencing mechanism

#### Analysis the influence of tourism industry on carbon emission and its influencing mechanism based on all samples

**Figure 2:**
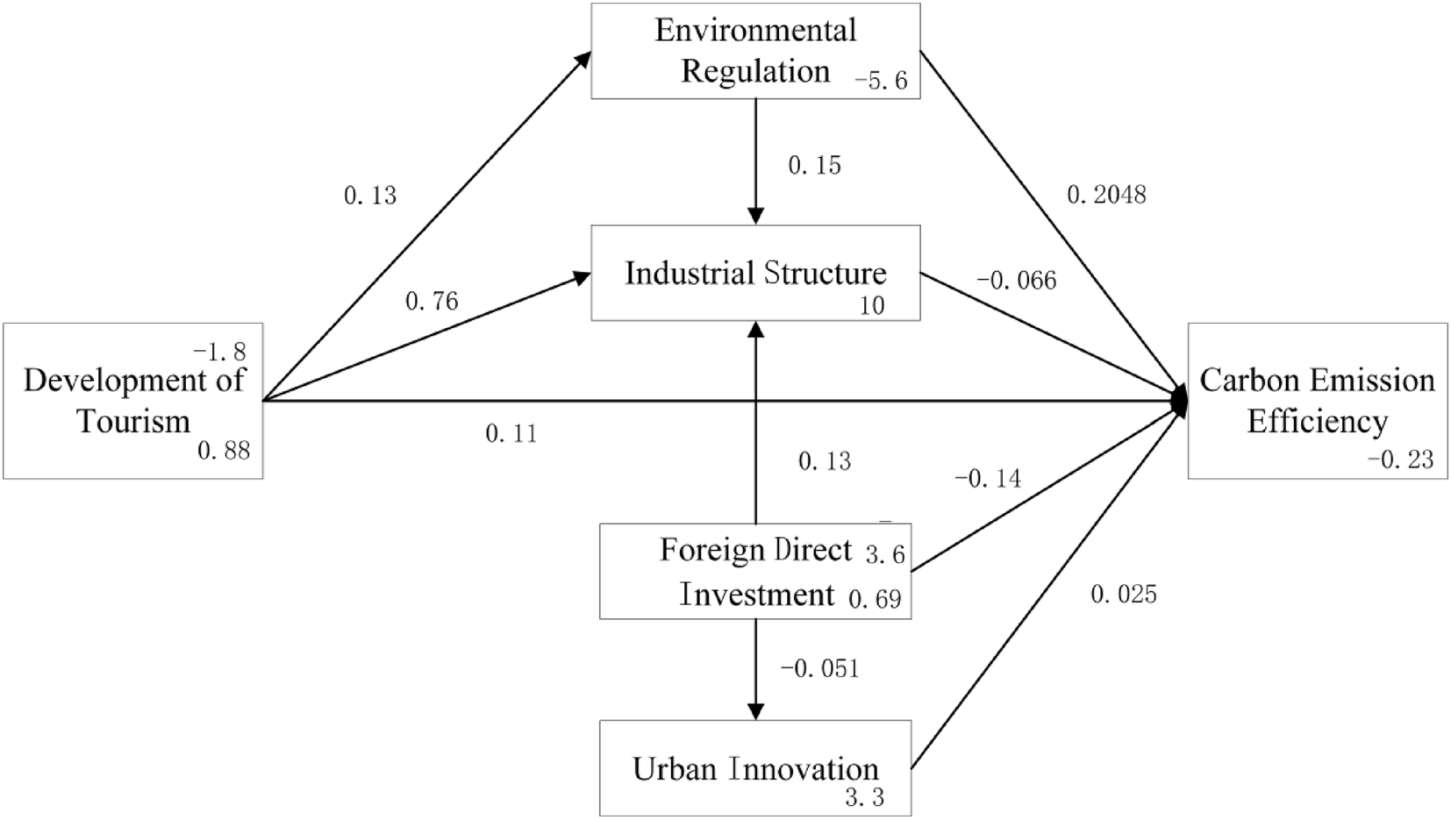
Structural equation model estimation results.

Based on the structural equation model, the required variables were introduced into the STATA software. The parameters of the constructed model were then estimated using the maximum likelihood estimation method, yielding the estimated results for standardized estimation coefficients, standard errors, Z-values, and P-values. The specific results are shown in Table 5 and Fig. 2.

**Table 5:** Maximum likelihood estimation results for structural equation models.

Path		Standard coefficient	Standard error	Z-value	*P* value
ln *CEE*	ln* tour*	0.1148***	0.0260	4.41	0.000
	ln *envi*	0.2048***	0.0424	7.58	0.000
	ln *ind*	0.0664**	0.0285	− 2.33	0.020
	ln *innov*	0.0248	0.0175	1.42	0.156
	ln *fdi*	− 0.1379***	0.0231	− 5.95	0.000
ln *envi*	ln *tour*	0 0.1280***	0 0.0191	6.69	0.000
ln *ind*	ln* tour*	0.7597***	0.0435	17.43	0.000
	ln *envi*	0.1524*	0.0907	1.68	0.093
	ln *fdi*	0.1292***	0.0475	2.72	0.007
ln *innov*	ln *fdi*	− 0.0514	0.0988	− 0.52	0.603
Variance	*e.* ln *CEE*	0.1885	0.0111		
	*e.* ln *envi*	0.1854	0.0109		
	*e.* ln *ind*	0.8793	0.0518		
	*e.* ln *innov*	3.8610	0.2275		

***, **, and *Significance levels of 1%, 5%, and 10%, respectively.

For all the tourist city samples, the structural equation model was estimated using the STATA software through the maximum likelihood estimation method. The estimated results included standardized estimation coefficients, standard errors, Z-values, and P-values. In terms of the overall fit of the model, the comparative fit index (CFI) is 0.902, slightly greater than 0.9, and the standardized residual root mean square (SRMR) is 0.07, slightly higher than 0.05 but less than 0.08 threshold, indicating that the overall fit of the model is good.

Logarithmic likelihood: − 8623.74;Likelihood ratio test of saturation model: chi-square(4) = 71.00, Prob > chi-square = 0.0000; The index of fit degree:RMSEA:0.109;AIC:14,115.030; BIC:16,023.930; CFI:0.902;SRMR: 0.070.

Table 5 and Fig. 2 demonstrate the mechanism of tourism's influence on carbon emission efficiency as follows: Firstly, a 1% increase in tourism development level leads to a direct increase of 0.1148% carbon emission efficiency, which passes the 1% significance level test. This indicates that the development of urban tourism significantly promotes the improvement in carbon emission efficiency. This indicates that tourism development can improve carbon emission efficiency, which is consistent with the study conducted by Si et al.^42^. On one hand, tourism stimulates local economic development,on the other hand, it consumes resources and emits less pollution compared to other industries. This implies that tourism development directly affects carbon emission efficiency and there is a mechanism of tourism → carbon emission efficiency. Secondly, the sustainable development of tourism imposes stricter demands on the ecological environment quality. As a result, the development of tourism prompts governments to introduce more rigorous environmental policies. The greater the intensity of urban environmental regulation, the more significant its impact on carbon emission efficiency. Each 1% increase in the level of tourism development would directly increase the intensity of environmental regulation by 0.1280%. There is a direct between environmental regulation and emission efficiency. Every 1% increase in environmental regulation, there is a corresponding 0.8% increase in carbon emission efficiency. This finding supports the conclusion that environmental regulation plays an effective role in reducing carbon emissions^43^. This finding also suggests that there is a mechanism of tourism → environmental regulation → carbon emission efficiency. Thirdly, empirical results reveal an influence effect of tourism → environmental regulation → industrial structure → carbon emission efficiency. Each 1% increase in environmental regulation would change the industrial structure by 0.1524%, and each 1% increase in industrial structure would increase carbon emission efficiency by 0.2048%. This suggests that the tourism industry impacts the local industrial structure by strengthening environmental regulations, thereby driving the improvement of carbon emission efficiency. Fourth, a 1% increase in tourism development level changes the industrial structure by 0.7597%, indicating that tourism development has a significant impact on the local industrial structure. Additionally, the estimated coefficient of industrial structure on carbon emission efficiency is 0.0664, meaning that the transformation of industrial structure promotes the improvement of carbon emission efficiency. In other words, tourism influences local carbon emission efficiency by influencing the industrial structure. There is a mechanism of tourism → industrial structure → carbon emission efficiency.

The mechanisms through which foreign direct investment influences carbon emission efficiency can be summarized in three aspects. Firstly, foreign direct investment has negative impacts on carbon emission efficiency. This indicates in tourist cities, FDI may intensify local energy consumption and production activities and becomes a refuge for heavily polluting enterprises. These findings are in line with the research conducted by Wang et al.^44^. Secondly, foreign direct investment significantly and positively affects the local industrial structure, indicating that the production technology brought by foreign direct investment has changed the industrial structure of the city. The results reveal an influence path of foreign direct investment → industrial structure. Empirical findings demonstrate that the industrial structure impacts carbon emission efficiency, resulting in a path of foreign direct investment → industrial structure → carbon emission efficiency. Thirdly, the impact of foreign direct investment on the innovation ability of cities did not pass the significance test (P = 0.603), indicating that there is no influence path of foreign direct investment → urban innovation → carbon emission efficiency.

#### Further analysis based on effect decomposition

Based on the above estimation results, this study further decomposed the direct, indirect, and total effects of each factor affecting carbon emission efficiency, the results are shown in Table 6.

**Table 6:** Direct and indirect effects of tourism on urban carbon emission efficiency.

Variable		Direct effect	Indirect effect	Total effect
ln *CEE*	ln* tour*	0.1148*** (0.000)	− 0.0524 (0.019)	0.0624** (0.040)
	ln *ind*	− 0.0664** (0.020)		− 0.6648** (0.020)
	ln *innov*	0.0248 (0.156)		0.0248 (0.156)
	ln *envi*	0.2048*** (0.000)	0.074 (0.173)	02,122*** (0.001)
	ln *fdi*	− 0.1379*** (0.000)	− 0.0098* (0.097)	− 0.1478*** (0.000)
ln *envi*	ln *tour*	0.1280*** (0.000)		0.1280*** (0.000)
	ln *envi*	0.1524* (0.093)		0.1524* (0.093)
ln *ind*	ln* tour*	0.7597*** (0.000)	0.0195 (0.103)	0.7792*** (0.000)
	ln *fdi*	0.1292** (0.007)		0.1292** (0.007)
ln *innov*	ln *fdi*	− 0.0514 (0.603)		− 0.0514 (0.603)

***, **, and *Significance levels of 1%, 5%, and 10%, respectively.

As shown in Table 6, the total effect of tourism on carbon emission efficiency is 0.0624, with a direct effect of 0.1148, accounting for 54.36% of the total effect. The direct effect passed the significance test but the indirect effect failed. This indicates that the influence of tourism on carbon emission mainly stems from the direct impact of tourism development on carbon emission efficiency, rather than the indirect effect. This empirical result aligns with the current reality in China. Cities can achieve the goal of reducing carbon emissions by focusing on green tourism and low-carbon tourism, promoting the use of environmentally friendly transportation modes in tourism, and improving the energy efficiency of tourism facilities. Furthermore, the estimates results reveal other important factors and pathways influencing carbon efficiency. Firstly, a higher intensity of environmental regulations can directly improve carbon emission efficiency. Every 1% increase in environmental regulation intensity would directly increase carbon emission efficiency by 0.2048%. Secondly, the direct effect of foreign direct investment on carbon emission efficiency is − 0.1379, and the indirect effect is − 0.0098, indicating that foreign direct investment has a negative impact on carbon emission efficiency in both direct and indirect aspects. Foreign investors may transfer polluting enterprises to tourist cities, resulting in increased carbon emissions and decreased carbon emission efficiency. Finally, changes in industrial structure have a positively effect on carbon emission efficiency. Every 1% change in industrial structure will reduce carbon emission efficiency by 0.0664%.

## Conclusion and discussion

Based on panel data from 31 tourist cities between 2005 and 2022, this study utilizes a structural equation model to analyze the influence of tourism on carbon emissions. The research findings indicate the following:

The carbon emission efficiency of tourism cities first decreased and then increased, reaching a peak of 0.923 in 2022. Second, tourism has a significant positive effect on carbon efficiency in the estimation of all samples. This influence can be summarized into three paths: tourism development → environmental regulation → carbon emission efficiency; Tourism development → environmental regulation → industrial structure → carbon emission efficiency; Tourism development → industrial structure → carbon emission efficiency. Thirdly, the influence of local tourism development on carbon emission efficiency mainly depends on the direct effect, which is consistent with the reality of China, and the development of tourism will also indirectly affect the local industrial structure. Environmental regulation also mainly depends on the direct effect on carbon emission efficiency, and foreign direct investment will lead to the reduction of carbon emission efficiency in both direct and indirect aspects.

Based on these research findings, this study proposes several suggestions: Firstly, tourism affects carbon emission efficiency through environmental regulation and industrial structure. To strengthen environmental regulation, local governments should increase supervision over enterprises, improve environmental standards, and take strict actions against environmental violations. These measures can enhance carbon emission efficiency and accelerate urban green transformation. Secondly, considering the negative impact of foreign direct investment on carbon emission efficiency, local governments should carefully evaluate potential environmental problems when dealing with foreign investments. Preferably, eco-friendly foreign direct investments should be prioritized. Thirdly, the influence of tourism on carbon emission efficiency mainly depends on the direct effect. Therefore, in the process of tourism development, the goal of improving carbon emission efficiency should be integrated to promote the development of tourism in the direction of eco-tourism and green tourism.

The content of this study is to analyze the influence of tourism on carbon efficiency, using 31 tourist cities as case studies. It introduces mechanism that explains how tourism development impacts carbon emission efficiency through considerations of environmental regulation and industrial structure. Nonetheless, it is important to acknowledge that this study also has certain limitations when compared to previous research. Firstly although this study considers the impact of environmental regulations on carbon emission efficiency, it did not conduct an in-depth analysis of different dimensions of environmental regulations. It is worth noting that the intensity and enforcement of environmental regulations may have significant differences in their impact on carbon emission efficiency as highlighted by Lin et al.^45^. Therefore, it is suggested that future studies incorporate the intensity and enforcement of environmental regulations into the model. By doing so, a more accurate assessment can be made regarding their impact on carbon efficiency. Secondly, this study suggests a pathway for tourism development to have an impact on carbon emission efficiency by influencing industrial structure. However, it does not delve deeply into the specific methods of adjusting industrial structure. Ahmad et al.^12^ have demonstrated that tourism's alternative impact on traditional manufacturing and high-carbon industries is a crucial approach to reducing carbon emissions. Future studies can potentially further analyze the contribution of tourism to the low-carbon transformation of industrial structure. Thirdly, this study suggests that FDI has a negative impact on carbon emission efficiency, but it does not fully discuss its potential positive effects. Zhang et al.^47^ have found that foreign direct investment can introduce advanced environmental protection technology and management experience, thereby improving the city's carbon emission efficiency. Therefore, future studies should how to achieve a positive impact on carbon emission efficiency through policy guidance and optimization of FDI structure.

## Data Availability

The data presented in this study are available on request from the corresponding authors.
